# Culture-based identification of pigmented *Porphyromonas* and *Prevotella* species in primary endodontic infections

**DOI:** 10.15171/joddd.2016.022

**Published:** 2016-08-17

**Authors:** Anuradha Rajaram, Vijayalakshmi S. Kotrashetti, Pradeep D. Somannavar, Preeti Ingalagi, Kishore Bhat

**Affiliations:** ^1^Postgraduate Student, Department of Oral Pathology and Microbiology, Maratha Mandal’s NG Halgekar Institute Dental Sciences and Research Centre, Belagavi -590010, Karnataka, India; ^2^Professor and HOD, Department of Oral Pathology and Microbiology, Maratha Mandal’s NG Halgekar Institute Dental Sciences and Research Centre, Belagavi -590010, Karnataka, India; ^3^Reader, Department of Oral Pathology and Microbiology, Maratha Mandal’s NG Halgekar Institute Dental Sciences and Research Centre, Belagavi -590010, Karnataka, India; ^4^Senior Lecturer, Department of Microbiology and Immunology, Maratha Mandal’s NG Halgekar Institute Dental Sciences and Research Centre, Belagavi -590010, Karnataka, India; ^5^Professor and HOD, Department of Microbiology and Immunology, Maratha Mandal’s NG Halgekar Institute Dental Sciences and Research Centre, Belagavi -590010, Karnataka, India

**Keywords:** Culture, infection, microorganism, Porphyromonas, Prevotella

## Abstract

***Background.*** The most common species isolated from primary endodontic infections are black-pigmented bacteria. These species are implicated in apical abscess formation due to their proteolytic activity and are fastidious in nature. Therefore, the present study was carried out to evaluate the presence and identification of various pigmented *Porphyromonas* and *Prevotella* species in the infected root canal through culture-based techniques.

***Methods.*** Thirty-one patients with primary endodontic infections were selected. Using sterile paper points, samples were collected from the root canals after access opening and prior to obturation, which were cultured using blood and kanamycin blood agar. Subsequently, biochemical test was used to identify the species and the results were analyzed using percentage comparison analysis, McNemar and chi-squared tests, Wilcoxon match pair test and paired t-test.

***Results.*** Out of 31 samples 26 were positive for black-pigmented organisms; the predominantly isolated species were *Prevotella* followed by *Porphyromonas*. In *Porphyromonas* only *P. gingivalis* was isolated. One of the interesting features was isolation of *P. gingivalis* through culture, which is otherwise very difficult to isolate through culture.

***Conclusion***. The presence of *Prevotella* and *Porphyromonas* species suggests that a significant role is played by these organisms in the pathogenesis of endodontic infections.

## Introduction

All the pathoses of the pulp and periapical tissues is caused by microorganisms. To effectively treat endodontic infections, clinicians must recognize the cause and effect of microbial invasion of the pulp space and surrounding periapical tissues.^1^ The belief of the microorganisms associated with endodontic disease requires the development of a basic understanding of the disease process and effective management of patients with endodontic infection.^1^

The root canal infection is primarily a dynamic process.^2^ The microbiota involved in the primary endodontic infection is dominated by anaerobic bacteria which have been cultivated by culture-dependent approaches.^3^

The most common species isolated from primary endodontic infections are black-pigmented bacteria.^2^These bacteria are obligate anaerobes, non-motile and non-sporing, which are fastidious and oxygen-sensitive organisms.^4^ These organisms are implicated in apical abscess formation due to their proteolytic activity. *Prevotella* species such as *P. intermedia* and *P. nigrescens* have been cultured from 26‒40% of infected root canals, suggesting an association with proteolytic activity.^2^

Due to their fastidious nature these organisms are difficult to culture. Many studies have been carried out with controversial results regarding the isolation of pigmented *Porphyromonas* and *Prevotella* species through culture.^5,6^

Culture, being considered the gold standard due to the identification of unexpected species, allows quantification of all the major viable microorganisms in the samples and broad range nature.^7^ Although molecular techniques appear to be pleasing and provide accurate results, it has certain limitation due to the chance of false positive results due to the detection of dead cells in the samples.^7^ Therefore, the present study was undertaken to review and detect black-pigmented organisms, mainly *Porphyromonas* and *Prevotella* species, in primary endodontic infections through a culture-based technique.

## Methods

Ethical clearance was obtained from the Institute’s Ethical Review Board. (Reference code of certificate: 36/2014/MMNGHIDS).

A total of 31 patients who attended the Department of Endodontics with the need for endodontic treatment due to infected root canals were included in this study.

The inclusion criteria were non-vital teeth presenting with a negative pulp test and deep carious lesion involving the pulp. All the permanent teeth having periapical radiolucency were included except for the third molars. The samples were obtained from single and multi-rooted teeth; in multi-rooted teeth, samples were collected from all the root canals which were non-vital. Exclusion criteria consisted of endodontically treated teeth, periodontal pocket >3 mm deep, periapical sinus/fistula, abnormal anatomy, calcified canals and any antibiotic therapy within the previous 3 months.

Patients who met both inclusion and exclusion criteria were enrolled for the study. Samples from the root canal were obtained after obtaining written informed consent forms from the patient. All the instruments and equipment were sterilized prior to the procedure. The procedure was carried by a single operator for all the 31 patients.

The samples were collected from root canals after rubber dam isolation from non-vital teeth using sterile paper points which were left in the canal for 60 seconds, after gaining access to the root canal using a sterile round bur and after gaining access to the root canal. Other steps such as cleaning, shaping and disinfection of the canal were carried out with 3% hydrogen peroxide and 2.5% sodium hypochlorite. Furthermore, another sample was collected prior to obturation, which was transferred to the laboratory in an appropriate transport medium (Reduced Transport Factor) immediately. The RTF samples were placed in a vortex (Biorad BR-2000 Vortexer) for 60 seconds and then centrifuged (500 rpm) for 1 minute. The deposits/sediments were cultured in blood agar enhanced with hemin and vitamin K and along with this in an anaerobic medium, i.e., Kanamycin blood agar. The culture plate was incubated in an anaerobic jar for 48‒72 hours at 37°C. After completion of the incubation, the plates were removed and observed for suspected colony characteristics (pigmentation and hemolysis) and simultaneous counting of such suspected colonies. Gram staining was performed using the suspected colonies for the gram-negative bacilli.

Identification of species was carried out by performing a biochemical test, which included catalase, indole and nitrate reductase along with sugar fermentation test using key sugars like glucose, sucrose, lactose, maltose, xylose, arabinose and salicin cellibiose. The indicator used for biochemical test was bromocresol purple which imparted a purple color to the solution. Purple indicates negative and yellow indicates positive for the particular sugar. A standard table^8^ was used to compare the results of biochemical test and identification of species was carried out. Statistical analysis was carried out by percentage comparison analysis. Further analyses for comparing access opening and pre-obturation samples were carried out using McNemar and chi-squared test and Wilcoxon match pair test. Colony forming units in all the samples were compared between access opening and pre-obturation samples using paired t-test.

## Results

**Figure 1.**
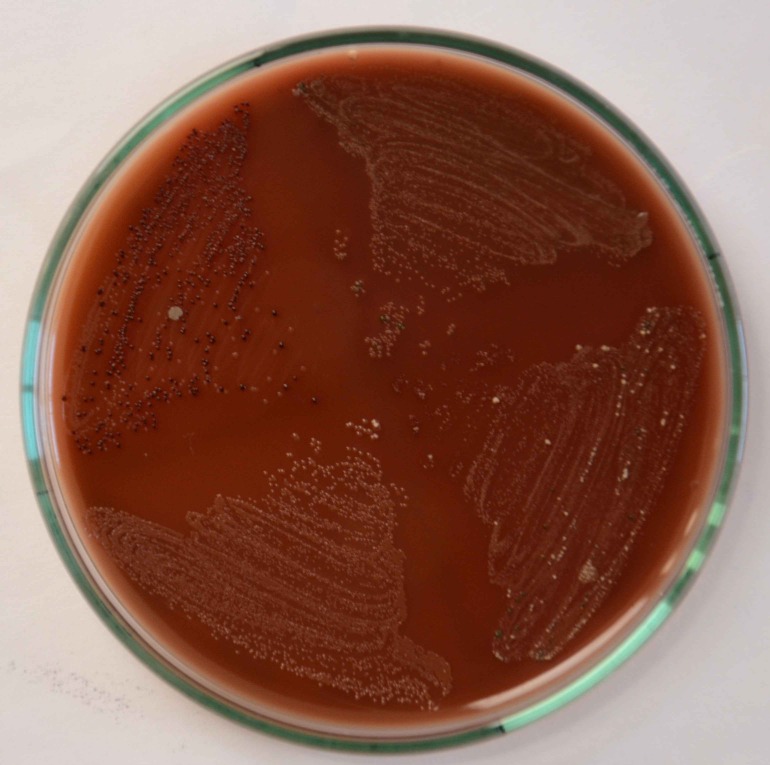
Photograph of culture plates showing growth of black-pigmented bacteria.

Of 31 samples 25 samples (80.6%) showed positivity for pigmented organisms in access opening samples and 5 samples showed positivity for pre-obturation cases (16.1%) (Figure 1). Of 31 patients, 16 were male and 15 were female. The patients’ ages were in the range of 18‒58 years with an average of 29.6 years.

Twenty-five cases which were positive during access opening showed maximum positivity for *Prevotella* species, followed by *Porphyromas* species. There were various subspecies of *Prevotella* which was isolated (Table 1). In pre-obturation samples, *Prevotella* species were dominant. In the 25 samples, 5 were anterior teeth and 20 were posterior teeth.

**Table 1:** Cases positive for species in access opening samples

**Serial**	**Species**	**No. of cases positive**
**1.**	*Porphyromonas gingivalis*	11
**2.**	*Prevotella loesschii*	5
**3.**	*Prevotella corporis*	2
**4.**	*Prevotella tannerae*	2
**5**	*Prevotella nigrescens*	2
**6.**	*Prevotella denticola*	1
**7.**	*Prevotella melaninogenica*	1
**8.**	*Prevotella intermedia*	1
**9.**	*Prevotella corporis/nigrescens*	1
**10.**	*Prevotella nigrescens/intermedia*	1
	Total cases positive for access opening -25	

In the anterior teeth the colony counts of *Porphyromonas gingivalis* were higher than *Prevotella* species (Table 2).

**Table 2:** Number of colony counts with species in the anterior teeth

**Serial. No.**	**Sample No.**	**Tooth**	**Species**	**Colony count (CFU/mL)**
**1.**	A-1	11	PG	1000
**2.**	A-3	22	*Prevotella loeschii*	1000
**3.**	A-6	12	PG	6000
**4.**	A-13	31	*Prevotella corporis*	4000
**5.**	A-53	12	*Prevotella loeschii*	1000
	Total No. of anterior teeth - 5			

In 20 posterior teeth, 9 teeth were positive for *Porphyromonas gingivalis* with maximum number of colony counts (Table 3). Statistically the access opening and pre-obturation samples were analyzed using McNemar test (P = 0.00002) and Wilcoxon match pair test (P = 0.0008), which showed significant results (Table 4). The colony forming units in the samples were compared between access opening and pre-obturation samples using paired t-test (P < 0.00001), which also showed significant results.

**Table 3:** Number of colony counts with species in the posterior teeth

**Serial. No**	**Sample No.**	**Tooth (FDI)**	**Species**	**Colony count** **( CFU/mL)**
**1.**	A-2	36	*PG*	1000
**2.**	A-5	26	*PG*	6000 20000
**3.**	A-9	36	*Prevotella corporis*	4000
**4.**	A-12	16	*PG*	2000
**5.**	A-15	15	*Prevotella tannerae*	8000
**6.**	A-16	16	*P. nigrescens*- 2 *P. tannerae* - 1	25000 15000 4000
**7.**	A-18	16		8000
**8.**	A-19	15	*PG*	15000
**9.**	A-20	26	*PG*	1000
**10.**	A-21	25	*P. intermedia*	25000
**11.**	A-22	*P. corporis*/*Nigrescens*	6000
**12.**	A-23	24	*P. nigrescens*/*intermedia*	34000
**13.**	A-26	16	*PG*	3000
**14.**	A-28	26	*PG*	4000
**15.**	A-30	14	*PG*	200000
**16.**	A-41	46	*P. melaninogenica*	20000
**17.**	A-42	45	*P. denticola*	12000
**18.**	A-59	14	*P. loeschii*	3000
**19.**	A-66	16	*P. loeschii*	30000
**20.**	A-69	26	*PG*	15000
	Total No. of posterior teeth -20			

**Table 4:** Comparison of access opening and pre-obturation samples

Access opening	Pre-obturation			
	Negative	Positive	Total	%
Negative	6	0	6	19.35
Positive	20	5	25	80.65
Total	26	5	31	100.00
%	83.87	16.13	100.00	
		McNemar/chi-squared=18.0501 , P=0.00002^*^		
		Wilcoxon matched pairs test, Z=3.9199, P=0.00008^*^		

^*^P <0.05

## Discussion

Pulp and periapical tissue pathoses are caused by microorganisms.^1^ Inflammatory reaction in the periapical tissue is due to the presence of bacteria within the root canal system. It is evident that an infected root canal system is a unique niche for the selective processes of microorganisms.^2^

Endodontic microflora has been the focus of considerable research over the years.^9^ Apical periodontitis with bone resorption will occur only in infected necrotic pulps and in cases in which microorganisms have survived in the apical portion of the root canal system and/or outside the apical foramen.^9^These factors are responsible for the treatment failures in the endodontic infection.^10^ Thus elimination of these microorganisms is of utmost importance to obtain root canal treatment success as endodontic infection is polymicrobial in nature.

Necrotic root canal environmental conditions are conducive to the establishment of microbiota conspicuously dominated by anaerobic bacteria.^11^

Black-pigmented bacteria are currently the focus of extensive basic science research and clinical studies as the main causative agent of endodontic infections, the severity of such infections and the resultant clinical symptoms associated with these infections.^12^ These anaerobic invaders have synergistic nature requiring specific nutrients for the growth and presence of other organisms to supply some of these products necessary for survival; therefore, they are not found clinically in pure cultures.^12^

Pathogenicity of bacteria is mainly related to the presence of lipopolysaccharides and peptidoglycans. They induce hormones like cytokines which play an important role in inflammation, stimulate B-lymphocytes, activate complement cascade, release various enzymes like collagenase, and enhance production of various pain mediators like bradykinin, histamine and prostaglandins; lipopolysaccharides (LPS) once released (as endotoxin) cause biological effects, including inflammation and bone resorption.^13^

The virulence potential of dark-pigmented bacteria has the ability to confront phagocytosis, degrade immunoglobulins and increase pathogenesis when in combination with other specific strains of bacteriodes.^13^ The majority of these organisms are fastidious in nature and difficult to isolate through culture. Thus the study was undertaken to isolate these microorganism through culture-based techniques.

In the current study the number of samples positive for black-pigmented organisms through culture in access opening samples was 80.6%, with 16.6% during pre-obturation. Various studies^6,14,15^ have shown a higher prevalence of black-pigmented species by PCR but these species can be efficiently identified through simple and cost-effective methods like culture.

In a study Gomes et al^16^ found that isolation of *Porphyromonas gingivalis* was rare through culture methods (1%) but it was the most commonly identified species by PCR because *Porphyromonas* species are oxygen-sensitive and fastidious microorganisms, which explains failure to culture these species, but in the present study we isolated *Porphyromonas gingivalis* in 44% of the access opening samples through culture. This suggests that if adequate precaution is taken and good anaerobic condition is maintained, *Porphyromonas* species can be cultivated through simple method as culture and biochemical assay. *Prevotella intermedia* and *Prevotella nigrescens*, which are not possible to be differentiated using culture, can be identified through PCR. In the present study *P. intermedia* (4%) and *P. nigrescens* (8%) were identified through culture in access opening samples.

The isolated black-pigmented organisms were compared to clinical signs and symptoms such as pain, tenderness to percussion and abscess, which were similar to the findings reported by Gomes et al,^16,17^ Griffee et al,^18^ Sundquist et al^19^ and Yoshida et al.^20^*Prevotella melaninogenica* isolated from access opening samples was significantly associated with clinical features such as foul odor, pain, sinus, swelling and pain on percussion. In our study, we isolated *P. melaninogenica* from 4% of access opening samples. *Prevotella intermedia* is associated with acute apical periodontitis. In our study, we could isolate *P. intermedia* (4%) in access opening samples.

*Porphyromonas gingivalis* showed the maximum colony counts in the anterior teeth (7000 CFU/mL) and the posterior teeth during access opening. *Porphyromonas gingivalis* produces the collagenase gene, enhancing its particular virulence.

*Prevotella loescheii* is associated with previous episodes of pain; in our study we could isolate 2 cases in anterior teeth and 2 cases in posterior teeth and made a distinction between anterior teeth and posterior teeth in relation to pigmented organisms during access opening but could not correlate them with respective teeth because of small sample size.

The colony count of *Porphyromonas gingivalis* (1000‒200000 CFU/mL) outnumbered *Prevotella* species. *Porphyromonas* species which are considered more virulent than *Prevotella* species and major pathogen in periodontitis are less frequent in acute endodontic infections but our findings showed *Porphyromonas gingivalis* was more virulent in endodontic infections with higher colony counts when compared to *Prevotella* species.

The five pre-obturation samples which were positive for *Prevotella nigrescens*, *Porphyromonas gingivalis*, *P. melaninogenica* and *P. corporis* could be due to the resistance of these organisms to biomechanical preparation. In these cases obturation should be referred to a later appointment because it may further lead to periapical infection such as periapical abscess formation.

## Conclusion

The presence of *Prevotella* and *Porphyromonas* species in the majority of samples shows that they play a significant role in the pathogenesis of endodontic infections. No other unusual pigmented species of *Prevotella* and *Porphyromonas* were identified through culture in this study. But we could isolate *Porphyromonas gingivalis* in greater numbers only through culture. This suggests that culture-based techniques can be effectively used for the isolation of these fastidious organisms in endodontic infection and can also be used as a cost-effective method in small laboratories.

## Acknowledgements

The authors would like to thank the HOD, staff and PGs of Conservative and Endodontics Department.

## Authors’ contributions

AR was responsible for the study design, data collection and compiling the manuscript**.** VSK was responsible for designing the research protocol and manuscript writing and reviewing**.** PDS was responsible for collecting review for the study. PI was responsible for designing the methodology and performing the analysis and data compilation. KB was responsible for review and editing the manuscript. All the authors have read and approved the final manuscript.

## Funding

The study was self-funded.

## Competing interests

The authors declare no competing interests with regards to the authorship and/or publication of this article.

## Ethics approval

This study was approved by Central Research and Ethics committee of Maratha Mandal’s NG Halgekar Institute of Dental Sciences and Research Centre (Code of ethical certificate No.36/2014/MMNGHIDS).
